# 5-Amino-1-(4-nitro­phen­yl)-1*H*-pyrazole-3-carbonitrile

**DOI:** 10.1107/S1600536811052147

**Published:** 2011-12-10

**Authors:** Qiang-Hua Jiang, Qiu He, Jian-Qiang Zhang, Yang Yang, Rong Wan

**Affiliations:** a Department of Applied Chemistry, College of Science, Nanjing University of Technology, No. 5 Xinmofan Road, Nanjing, Nanjing 210009, People’s Republic of China

## Abstract

The title compound, C_10_H_7_N_5_O_2_, was synthesized by the reaction of 4-nitro­aniline and 2,3-dicyano­propionic acid ethyl ester. In the crystal, N—H⋯O and C—H⋯O hydrogen bonds link the mol­ecules, forming a three-dimensional network.

## Related literature

*N*-pyrazole derivatives are of great inter­est because of their chemical and pharmaceutical properties, see: Cheng *et al.* (2008 ▶). They also exhibit diverse biological activity such as insecticidal (Zhao *et al.*, 2010 ▶) and anti­fungal activities (Liu *et al.*, 2010 ▶). For bond-length data, see: Allen *et al.* (1987 ▶).
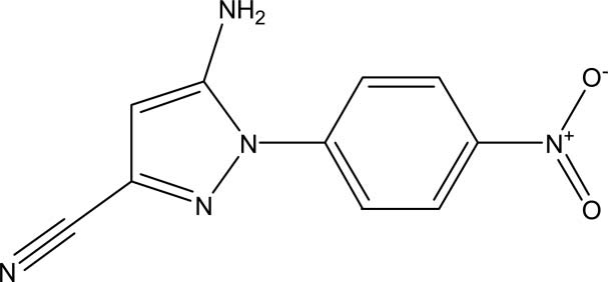


## Experimental

### 

#### Crystal data

C_10_H_7_N_5_O_2_
*M* *_r_* = 229.21
Monoclinic, 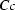

*a* = 3.7685 (2) Å
*b* = 27.3441 (17) Å
*c* = 10.1294 (8) Å
β = 96.20 (3)°
*V* = 1037.70 (12) Å^3^
*Z* = 4
Mo *K*α radiation
μ = 0.11 mm^−1^
*T* = 293 K
0.30 × 0.30 × 0.10 mm

#### Data collection

Enaf–Nonius CAD-4 diffractometer
Absorption correction: ψ scan (North *et al.*, 1968 ▶) *T*_min_ = 0.968, *T*_max_ = 0.989
2148 measured reflections
951 independent reflections
856 reflections with *I* > 2σ(*I*)
*R*_int_ = 0.071
3 standard reflections every 200 reflections  intensity decay: 1%

#### Refinement

*R*[*F* ^2^ > 2σ(*F* ^2^)] = 0.048
*wR*(*F* ^2^) = 0.123
*S* = 1.00
951 reflections
155 parameters
2 restraints
H-atom parameters constrained
Δρ_max_ = 0.26 e Å^−3^
Δρ_min_ = −0.31 e Å^−3^

### 

Data collection: *CAD-4 Software* (Enraf–Nonius, 1989 ▶); cell refinement: *CAD-4 Software*; data reduction: *XCAD4* (Harms & Wocadlo, 1995 ▶); program(s) used to solve structure: *SHELXS97* (Sheldrick, 2008 ▶); program(s) used to refine structure: *SHELXL97* (Sheldrick, 2008 ▶); molecular graphics: *SHELXTL* (Sheldrick, 2008 ▶); software used to prepare material for publication: *SHELXL97*.

## Supplementary Material

Crystal structure: contains datablock(s) global, I. DOI: 10.1107/S1600536811052147/hg5146sup1.cif

Structure factors: contains datablock(s) I. DOI: 10.1107/S1600536811052147/hg5146Isup2.hkl

Supplementary material file. DOI: 10.1107/S1600536811052147/hg5146Isup3.cml

Additional supplementary materials:  crystallographic information; 3D view; checkCIF report

## Figures and Tables

**Table 1:** Hydrogen-bond geometry (Å, °)

*D*—H⋯*A*	*D*—H	H⋯*A*	*D*⋯*A*	*D*—H⋯*A*
N4—H4*B*⋯O2^i^	0.86	2.53	3.335 (5)	156
C4—H4*A*⋯O1^ii^	0.93	2.52	3.216 (5)	132

Symmetry codes: (i) 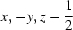; (ii) 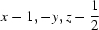.

**Fig. 1.**
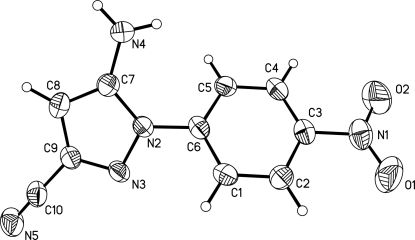
The molecular structure of the title molecule, with the atom-numbering scheme. Displacement ellipsoids are drawn at the 50% probability level.

**Fig. 2.**
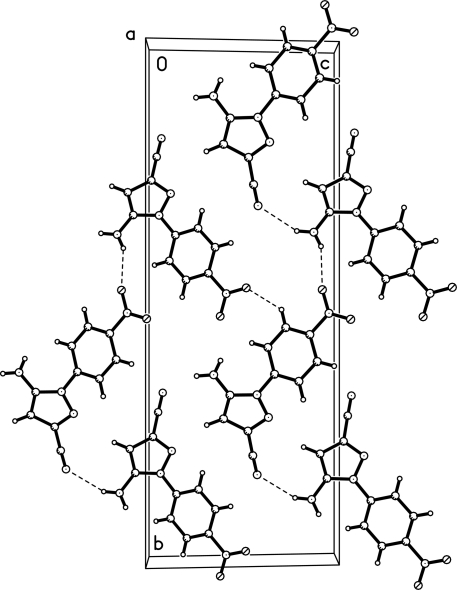
A partial packing diagram for (I). Hydrogen bonds are shown as dashed lines.

## supplementary crystallographic information

### Comment

In a variety of biological heterocyclic compounds, *N*-pyrazole derivatives are of great interest because of their chemical and pharmaceutical properties (Cheng *et al.*, 2008). These compounds are known to exhibit diverse biological activities, such as insecticidal (Zhao *et al.*, 2010) and antifungal activities (Liu *et al.*, 2010).

Here we report the crystal structure of the title compound,(I). In the molecule of the title compound (Fig.1),the bond lengths and angles are within normal ranges (Allen *et al.*, 1987). Rings A(C1—C6),B(N2/N3/C9/C8/C7) are, of course, planar. The dihedral angle between them is A/B = 34.3 (1) Å. In the crystal structure, intermolecular N—H···O and C—H···O hydrogen bonds (Table 1) link the molecules to form a three-dimensional network (Fig. 2), in which they may be effective in the stabilization of the structure.

### Experimental

Sodium nitrite(1.49 g) was dissolved 10 ml water, then the solution was added dropwise to a mixture of 4-nitrophenylamino (0.02 mol) and 36.5% aq. HCl(5 ml) at 0–5°C. After the addition, the above reaction mixture was stirred for 10 min at 0–5°C. 2,3-Dicyano-propionic acid ethyl ester (0.02 mol) was added dropwise and stirred for 2 hr at room temperature. The reaction mixture was extracted with dichloromethane and the pH was adjusted to 9 with ammonia. The aqueous layer was removed and the organic layer was dried over anhydrous Na_2_SO_4_, concentrated and precipitated. The pure compound (I) was obtained by recrystallization from ethanol. Crystals of (I) suitable for X-ray diffraction were obtained by slow evaporation of an acetone solution.

### Refinement

All H atoms were positioned geometrically, with N—H = 0.86 Å and C—H = 0.93Å, and constrained to ride on their parent atoms, with *U*_iso_(H) = 1.2*U*_eq_(C, N).

### Crystal data

**Table:**

C_10_H_7_N_5_O_2_	*F*(000) = 472
*M**_r_* = 229.21	*D*_x_ = 1.467 Mg m^−^^3^
Monoclinic, *C**c*	Melting point = 498–501 K
Hall symbol: C -2yc	Mo *K*α radiation, λ = 0.71073 Å
*a* = 3.7685 (2) Å	Cell parameters from 25 reflections
*b* = 27.3441 (17) Å	θ = 9–13°
*c* = 10.1294 (8) Å	µ = 0.11 mm^−^^1^
β = 96.20 (3)°	*T* = 293 K
*V* = 1037.70 (12) Å^3^	Block, yellow
*Z* = 4	0.30 × 0.30 × 0.10 mm

### Data collection

**Table:**

Enaf–Nonius CAD-4 diffractometer	856 reflections with *I* > 2σ(*I*)
Radiation source: fine-focus sealed tube	*R*_int_ = 0.071
graphite	θ_max_ = 25.4°, θ_min_ = 2.5°
ω/2θ scans	*h* = 0→4
Absorption correction: ψ scan (North *et al.*, 1968)	*k* = −32→32
*T*_min_ = 0.968, *T*_max_ = 0.989	*l* = −12→12
2148 measured reflections	3 standard reflections every 200 reflections
951 independent reflections	intensity decay: 1%

### Refinement

**Table:**

Refinement on *F*^2^	Secondary atom site location: difference Fourier map
Least-squares matrix: full	Hydrogen site location: inferred from neighbouring sites
*R*[*F*^2^ > 2σ(*F*^2^)] = 0.048	H-atom parameters constrained
*wR*(*F*^2^) = 0.123	*w* = 1/[σ^2^(*F*_o_^2^) + (0.093*P*)^2^] where *P* = (*F*_o_^2^ + 2*F*_c_^2^)/3
*S* = 1.00	(Δ/σ)_max_ < 0.001
951 reflections	Δρ_max_ = 0.26 e Å^−^^3^
155 parameters	Δρ_min_ = −0.31 e Å^−^^3^
2 restraints	Extinction correction: *SHELXL97* (Sheldrick, 2008), Fc^*^=kFc[1+0.001xFc^2^λ^3^/sin(2θ)]^-1/4^
Primary atom site location: structure-invariant direct methods	Extinction coefficient: 0.017 (6)

### Special details

**Table:**

Geometry. All e.s.d.'s (except the e.s.d. in the dihedral angle between two l.s. planes) are estimated using the full covariance matrix. The cell e.s.d.'s are taken into account individually in the estimation of e.s.d.'s in distances, angles and torsion angles; correlations between e.s.d.'s in cell parameters are only used when they are defined by crystal symmetry. An approximate (isotropic) treatment of cell e.s.d.'s is used for estimating e.s.d.'s involving l.s. planes.
Refinement. Refinement of *F*^2^ against ALL reflections. The weighted *R*-factor *wR* and goodness of fit *S* are based on *F*^2^, conventional *R*-factors *R* are based on *F*, with *F* set to zero for negative *F*^2^. The threshold expression of *F*^2^ > σ(*F*^2^) is used only for calculating *R*-factors(gt) *etc*. and is not relevant to the choice of reflections for refinement. *R*-factors based on *F*^2^ are statistically about twice as large as those based on *F*, and *R*- factors based on ALL data will be even larger.

### Fractional atomic coordinates and isotropic or equivalent isotropic displacement parameters (Å^2^)

**Table:**

	*x*	*y*	*z*	*U*_iso_*/*U*_eq_	
C1	0.6000 (9)	0.14112 (13)	0.7685 (3)	0.0345 (8)	
H1A	0.6497	0.1737	0.7890	0.041*	
N1	0.7783 (9)	0.01800 (13)	0.9127 (4)	0.0495 (9)	
O1	0.9593 (11)	0.02857 (14)	1.0134 (4)	0.0782 (13)	
N2	0.2632 (8)	0.16762 (10)	0.5653 (3)	0.0343 (7)	
C2	0.7254 (10)	0.10486 (14)	0.8562 (4)	0.0370 (8)	
H2A	0.8616	0.1124	0.9357	0.044*	
O2	0.6973 (13)	−0.02441 (11)	0.8819 (4)	0.0763 (12)	
C3	0.6406 (9)	0.05648 (13)	0.8214 (3)	0.0347 (8)	
N3	0.1670 (9)	0.21026 (11)	0.6233 (3)	0.0362 (7)	
C4	0.4432 (9)	0.04433 (12)	0.7061 (4)	0.0361 (8)	
H4A	0.3940	0.0117	0.6858	0.043*	
N4	0.2620 (12)	0.13064 (12)	0.3498 (3)	0.0517 (9)	
H4B	0.3581	0.1043	0.3834	0.062*	
H4C	0.2109	0.1331	0.2652	0.062*	
C5	0.3155 (9)	0.08073 (14)	0.6189 (3)	0.0353 (8)	
H5A	0.1748	0.0731	0.5405	0.042*	
N5	−0.2189 (12)	0.32149 (13)	0.5701 (4)	0.0571 (10)	
C6	0.4020 (9)	0.12921 (12)	0.6510 (4)	0.0316 (7)	
C7	0.1906 (10)	0.16856 (13)	0.4306 (4)	0.0367 (8)	
C8	0.0411 (12)	0.21334 (14)	0.3985 (4)	0.0412 (8)	
H8A	−0.0359	0.2255	0.3146	0.049*	
C9	0.0310 (9)	0.23653 (12)	0.5213 (4)	0.0347 (8)	
C10	−0.1059 (10)	0.28392 (14)	0.5470 (4)	0.0411 (9)	

### Atomic displacement parameters (Å^2^)

**Table:**

	*U*^11^	*U*^22^	*U*^33^	*U*^12^	*U*^13^	*U*^23^
C1	0.0409 (17)	0.0288 (16)	0.0351 (16)	−0.0030 (14)	0.0102 (15)	−0.0032 (14)
N1	0.050 (2)	0.0475 (19)	0.053 (2)	0.0072 (16)	0.0127 (18)	0.0160 (16)
O1	0.094 (3)	0.070 (2)	0.065 (2)	0.005 (2)	−0.021 (2)	0.0227 (19)
N2	0.0461 (16)	0.0256 (14)	0.0317 (14)	0.0020 (13)	0.0072 (12)	0.0000 (12)
C2	0.0424 (18)	0.0389 (18)	0.0305 (16)	0.0005 (16)	0.0073 (14)	0.0026 (14)
O2	0.111 (3)	0.0379 (17)	0.079 (2)	0.0041 (19)	0.010 (2)	0.0194 (16)
C3	0.0330 (17)	0.0337 (17)	0.0394 (17)	0.0046 (15)	0.0129 (15)	0.0066 (15)
N3	0.0488 (17)	0.0255 (14)	0.0357 (15)	0.0032 (12)	0.0108 (13)	−0.0018 (10)
C4	0.0400 (19)	0.0275 (17)	0.0427 (18)	0.0031 (14)	0.0135 (16)	0.0008 (14)
N4	0.090 (3)	0.0352 (16)	0.0300 (14)	0.0101 (17)	0.0084 (16)	−0.0028 (12)
C5	0.0416 (19)	0.0306 (18)	0.0340 (17)	−0.0001 (14)	0.0053 (15)	−0.0029 (12)
N5	0.080 (3)	0.0384 (18)	0.055 (2)	0.0178 (19)	0.0208 (19)	0.0037 (16)
C6	0.0351 (16)	0.0261 (15)	0.0360 (17)	0.0022 (14)	0.0145 (14)	0.0033 (13)
C7	0.0441 (19)	0.0337 (17)	0.0332 (16)	−0.0010 (15)	0.0091 (15)	0.0011 (14)
C8	0.050 (2)	0.0407 (18)	0.0330 (17)	0.0015 (16)	0.0047 (15)	0.0086 (14)
C9	0.0379 (18)	0.0289 (17)	0.0383 (17)	0.0014 (15)	0.0090 (14)	0.0023 (13)
C10	0.049 (2)	0.036 (2)	0.0410 (19)	0.0059 (17)	0.0150 (17)	0.0075 (15)

### Geometric parameters (Å, °)

**Table:**

C1—C6	1.374 (5)	C4—C5	1.382 (5)
C1—C2	1.381 (5)	C4—H4A	0.9300
C1—H1A	0.9300	N4—C7	1.366 (5)
N1—O1	1.199 (5)	N4—H4B	0.8600
N1—O2	1.231 (5)	N4—H4C	0.8600
N1—C3	1.459 (5)	C5—C6	1.395 (5)
N2—C7	1.363 (5)	C5—H5A	0.9300
N2—N3	1.371 (4)	N5—C10	1.146 (5)
N2—C6	1.425 (4)	C7—C8	1.372 (5)
C2—C3	1.397 (5)	C8—C9	1.400 (5)
C2—H2A	0.9300	C8—H8A	0.9300
C3—C4	1.357 (5)	C9—C10	1.429 (5)
N3—C9	1.316 (5)		
			
C6—C1—C2	120.2 (3)	C7—N4—H4B	120.0
C6—C1—H1A	119.9	C7—N4—H4C	120.0
C2—C1—H1A	119.9	H4B—N4—H4C	120.0
O1—N1—O2	123.1 (4)	C4—C5—C6	118.6 (3)
O1—N1—C3	119.7 (4)	C4—C5—H5A	120.7
O2—N1—C3	117.2 (4)	C6—C5—H5A	120.7
C7—N2—N3	112.3 (3)	C1—C6—C5	121.2 (3)
C7—N2—C6	130.1 (3)	C1—C6—N2	118.8 (3)
N3—N2—C6	117.6 (3)	C5—C6—N2	119.8 (3)
C1—C2—C3	117.7 (3)	N2—C7—N4	123.7 (3)
C1—C2—H2A	121.2	N2—C7—C8	106.7 (3)
C3—C2—H2A	121.2	N4—C7—C8	129.6 (4)
C4—C3—C2	122.6 (3)	C7—C8—C9	104.1 (3)
C4—C3—N1	119.6 (3)	C7—C8—H8A	128.0
C2—C3—N1	117.8 (3)	C9—C8—H8A	128.0
C9—N3—N2	103.1 (3)	N3—C9—C8	113.9 (3)
C3—C4—C5	119.6 (3)	N3—C9—C10	118.0 (3)
C3—C4—H4A	120.2	C8—C9—C10	128.2 (3)
C5—C4—H4A	120.2	N5—C10—C9	178.4 (4)
			
C6—C1—C2—C3	−0.6 (5)	C4—C5—C6—N2	−177.7 (3)
C1—C2—C3—C4	0.1 (5)	C7—N2—C6—C1	150.6 (4)
C1—C2—C3—N1	178.9 (3)	N3—N2—C6—C1	−33.0 (5)
O1—N1—C3—C4	178.4 (4)	C7—N2—C6—C5	−33.7 (6)
O2—N1—C3—C4	−2.3 (5)	N3—N2—C6—C5	142.6 (3)
O1—N1—C3—C2	−0.4 (5)	N3—N2—C7—N4	179.8 (4)
O2—N1—C3—C2	178.9 (4)	C6—N2—C7—N4	−3.7 (6)
C7—N2—N3—C9	0.8 (4)	N3—N2—C7—C8	−0.1 (4)
C6—N2—N3—C9	−176.1 (3)	C6—N2—C7—C8	176.4 (4)
C2—C3—C4—C5	−0.7 (5)	N2—C7—C8—C9	−0.7 (4)
N1—C3—C4—C5	−179.4 (3)	N4—C7—C8—C9	179.4 (4)
C3—C4—C5—C6	1.6 (5)	N2—N3—C9—C8	−1.3 (4)
C2—C1—C6—C5	1.6 (5)	N2—N3—C9—C10	178.7 (3)
C2—C1—C6—N2	177.2 (3)	C7—C8—C9—N3	1.3 (5)
C4—C5—C6—C1	−2.1 (5)	C7—C8—C9—C10	−178.7 (4)

### Hydrogen-bond geometry (Å, °)

**Table:**

*D*—H···*A*	*D*—H	H···*A*	*D*···*A*	*D*—H···*A*
N4—H4B···O2^i^	0.86	2.53	3.335 (5)	156.
C4—H4A···O1^ii^	0.93	2.52	3.216 (5)	132.

Symmetry codes: (i) *x*, −*y*, *z*−1/2; (ii) *x*−1, −*y*, *z*−1/2.
